# Preterm birth accelerates the maturation of spontaneous and resting activity in the visual cortex

**DOI:** 10.3389/fnint.2023.1149159

**Published:** 2023-05-15

**Authors:** Isabelle F. Witteveen, Emily McCoy, Troy D. Holsworth, Catherine Z. Shen, Winnie Chang, Madelyn G. Nance, Allison R. Belkowitz, Avery Dougald, Meghan H. Puglia, Adema Ribic

**Affiliations:** ^1^Department of Psychology, College and Graduate School of Arts and Sciences, University of Virginia, Charlottesville, VA, United States; ^2^Program in Fundamental Neuroscience, University of Virginia, Charlottesville, VA, United States; ^3^Department of Neurology, School of Medicine, University of Virginia, Charlottesville, VA, United States

**Keywords:** preterm (birth), neurodevelopment, visual activity, inhibition, EEG, neuron, aperiodic component, resting activity

## Abstract

Prematurity is among the leading risks for poor neurocognitive outcomes. The brains of preterm infants show alterations in structure and electrical activity, but the underlying circuit mechanisms are unclear. To address this, we performed a cross-species study of the electrophysiological activity in the visual cortices of prematurely born infants and mice. Using electroencephalography (EEG) in a sample of healthy preterm (*N* = 29) and term (*N* = 28) infants, we found that the maturation of the aperiodic EEG component was accelerated in the preterm cohort, with a significantly flatter 1/f slope when compared to the term infants. The flatter slope was a result of decreased spectral power in the theta and alpha bands and was correlated with the degree of prematurity. To determine the circuit and cellular changes that potentially mediate the changes in 1/f slope after preterm birth, we used *in vivo* electrophysiology in preterm mice and found that, similar to infants, preterm birth results in a flattened 1/f slope. We analyzed neuronal activity in the visual cortex of preterm (*N* = 6) and term (*N* = 9) mice and found suppressed spontaneous firing of neurons. Using immunohistochemistry, we further found an accelerated maturation of inhibitory circuits. In both preterm mice and infants, the functional maturation of the cortex was accelerated, underscoring birth as a critical checkpoint in cortical maturation. Our study points to a potential mechanism of preterm birth-related changes in resting neural activity, highlighting the utility of a cross-species approach in studying the neural circuit mechanisms of preterm birth-related neurodevelopmental conditions.

## Introduction

Preterm infants born < 37 weeks’ gestation currently represent a tenth of the infant population, with rising prevalence. Risks for prematurity include multiple gestations and pregnancy complications, with health outcomes positively correlated with increasing age at birth (Blencowe et al., 2012, 2013; Barfield, 2018). As the brain is still developing during the third postnatal month, premature birth can interrupt the trajectory of multiple developmental processes in the brain, resulting in neurological conditions, developmental delays, and visual deficits (Limperopoulos et al., 2007; Mann et al., 2011; Ortinau and Neil, 2015; Leung et al., 2018). Neurocognitive and behavioral conditions are common in preterm children and adults, with a high prevalence of attention disorders and autism spectrum disorder (Limperopoulos et al., 2007; Strang-Karlsson et al., 2010; Jaekel et al., 2013; Treyvaud et al., 2013; Del Hoyo Soriano et al., 2020; Crump et al., 2021; McGowan and Sheinkopf, 2021; Shuffrey et al., 2022).

At birth, the brains of preterm infants are significantly smaller and often display persistent abnormalities in functional connectivity (Pandit et al., 2014; Bouyssi-Kobar et al., 2018, 2016; Hunt et al., 2019; Tokariev et al., 2019). Poor neurocognitive outcomes are, in some cases, associated with reduced connectivity in the brains of preterm infants and children (Ball et al., 2013; Pandit et al., 2014; Wehrle et al., 2018; Kozhemiako et al., 2019; Jakab et al., 2020). On the other hand, sensory cortices of preterm infants show increased connectivity when compared to *in utero* fetuses of the same gestational age, suggesting that preterm birth alters developmental trajectories in a circuit-specific manner (De Asis-Cruz et al., 2020). Given the increasing prevalence of preterm birth, identifying the changes in brain structure and function resulting from preterm birth, as well as their mechanisms, is vital for understanding the drivers of poor neurocognitive outcomes in the preterm population.

Electroencephalography (EEG) has been increasingly used in the clinical setting both for prognostic and diagnostic purposes (Holmes and Lombroso, 1993; Watanabe et al., 1999; McCoy and Hahn, 2013; Rivera et al., 2021). EEG is non-invasive and as little as 40 s of recording is sufficient to construct basic measures of neural activity, such as neural oscillations (periodic) and background (aperiodic) neural activity (O’Toole and Boylan, 2019). Both periodic and aperiodic components of the EEG power spectra undergo developmental changes, with spectral power decreasing in low frequency power bands and the slope of aperiodic component flattening with increasing age (Gasser et al., 1988; Saby and Marshall, 2012; Schaworonkow and Voytek, 2021). Both components have been used as a diagnostic and prognostic tool in the preterm population, with varying success (Watanabe et al., 1999; Vanhatalo et al., 2002; O’Toole and Boylan, 2019; Lloyd et al., 2021; Nishiyori et al., 2021; Nordvik et al., 2022; Shuffrey et al., 2022). Early postnatal EEG correlates with neurocognitive outcomes in childhood and the aperiodic component is associated with autism risk (Cainelli et al., 2021; Nordvik et al., 2022; Shuffrey et al., 2022). It is currently unknown how preterm birth affects EEG components in healthy preterm born infants when compared to corrected age-matched term infants.

Increasing use of animal models that replicate electrophysiological signatures of preterm birth-related brain injury, such as hypoxia, has aided in identifying the cellular and circuit changes after preterm birth (El-Hayek et al., 2011; Zanelli et al., 2014; Burnsed et al., 2019; Johnson et al., 2022). Very early preterm infants and hypoxic mice share common neural deficits, such as impaired development and integration of cortical interneurons (Malik et al., 2013; Lacaille et al., 2019; Stolp et al., 2019; Scheuer et al., 2021). Animal models of preterm birth itself are less commonly studied, likely due to low viability (Dudley et al., 1996; Elovitz and Mrinalini, 2004; McCarthy et al., 2018). Previous research has shown that preterm mice do not display impairments in tasks assaying anxiety-like and social behaviors, or in basal synaptic transmission in the hippocampus (Chiesa et al., 2019). However, impairments of visual processing and visuo-spatial attention are more common in preterm children (Fazzi et al., 2012; Leung et al., 2018; Burstein et al., 2021; Chokron et al., 2021), but it is currently unknown how prematurity affects the activity in the primary visual areas. An animal model that replicates the effects of prematurity on neural activity, especially in sensory areas, would be crucial for identifying the drivers of poor neurodevelopmental outcomes in preterm population.

In this study, we used a cross-species approach to determine the effects of premature birth on neural activity in infants and mice at a network level, and then at a circuit and cellular level in preterm born mice. We used EEG in healthy preterm infants and *in vivo* electrophysiology in preterm mice to identify changes in periodic and aperiodic neural activity associated with prematurity. We focused our study on the visual cortex for three reasons: (1) it is well characterized anatomically and functionally in both humans and mice (Huttenlocher et al., 1982; Norcia et al., 1987; Antonini et al., 1999; Colonnese et al., 2010; Pinto et al., 2010; Lunghi et al., 2011; Haak et al., 2015; Kiorpes, 2016; Shen and Colonnese, 2016; Mitchell and Maurer, 2022), (2) previous research suggests that the maturation of visual areas is accelerated after preterm birth (Jandó et al., 2012; Schwindt et al., 2018; De Asis-Cruz et al., 2020), and (3) the aforementioned incidence of visual impairments in preterm born children. We find that the aperiodic 1/f component slope is significantly flatter in both preterm infants and mice, confirming accelerated maturation of visual areas. Using preterm mice, we identify increased inhibition in the preterm brain as a potential mechanism of preterm birth-related changes in neural activity.

## Materials and methods

### Infants

Sixty-eight preterm infants recruited from the University of Virginia neonatal intensive care unit (NICU) and 75 term infants recruited from the greater Charlottesville area completed a resting-state EEG paradigm as part of a larger, ongoing study. Inclusion criteria for the preterm sample were birth prior to 37 weeks’ gestation, corrected age 0–4 months at testing, English-speaking legal guardian, no known uncorrectable severe auditory or visual deficit, and health condition deemed sufficiently stable for participation by the neonatology care team. Inclusion criteria for the term sample were birth after 37 weeks’ gestation, age 0–4 months at testing, English-speaking legal guardian, no known family history of neurodevelopmental disorder, and no known uncorrectable auditory or visual deficit. All data were collected at the onset of the critical period for the development of binocularity (after birth) (Fawcett et al., 2005), a visual function that is highly sensitive to altered perinatal experience (Freeman and Ohzawa, 1992; Fawcett et al., 2005; Jandó et al., 2012). The infant’s legal guardian provided written informed consent for a protocol approved by the University of Virginia (UVA) Institutional Review Board (HSR210330 or HSR19514, principal investigator: Puglia). Families were compensated $50 for their participation.

### EEG acquisition and preprocessing

**FIGURE 1:**
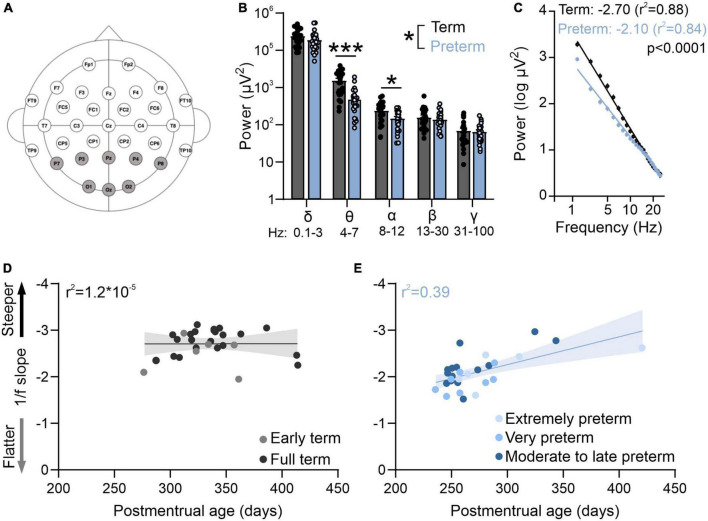
Preterm humans display flatter slope of aperiodic EEG component. **(A)** EEG cap montage. Highlighted parietal and occipital channels are analyzed. **(B)** Preterm birth significantly reduces resting theta and alpha band power. Two-way ANOVA interaction: *p* = 0.039; *F*(4,220) = 2.55; *N* = 29 preterm and 28 term-born infants. Sidak’s multiple comparisons test: theta *p* < 0.0001, alpha *p* = 0.022. **(C)** Preterm infants have a significantly flatter slope of log transformed 2–25 Hz power. Linear regression; slope and *r*^2^ values are indicated. Data shown as mean or mean ± SEM. **(D)** 1/f slope is not correlated with postmenstrual age in term infants. **(E)** 1/f slope values are significantly correlated with postmenstrual age in preterm infants (*r*^2^ = 0.39, *p* = 0.01). *p ≤ 0.05; ***p ≤ 0.0001.

Full scalp resting-state EEG (Figure 1A) was recorded while the infant rested in a caregiver’s arms for up to 7 min. Data were preprocessed with an automated preprocessing pipeline specifically validated and shown to generate reliable estimates for the computation of aperiodic signal in pediatric EEG data (APPLESED, Puglia et al., 2022). See Supplementary material for additional details. After preprocessing, 29 preterm and 28 term infants had sufficient data for subsequent analysis. See Table 1 for participant demographic and perinatal characteristics.

**TABLE 1:** Participant demographic and perinatal characteristics.

	Preterm infants (*n* = 29)	Term infants (*n* = 28)
GA (weeks), mean ± SD	31.4 ± 3.4	39.2 ± 0.9
Extremely preterm (<28 weeks)	6 (20.7%)	−
Very preterm (28–32 weeks)	8 (27.6%)	−
Moderate preterm (32–34 weeks)	4 (13.8%)	−
Late preterm (34–37 weeks)	11 (37.9%)	−
Early term (37–39 weeks)	−	6 (21.4%)
Full term (39–41 weeks)	−	22 (78.6%)
Late term (>41 weeks)	−	−
PMA at test (weeks), mean ± SD	39.0 ± 5.4	47.9 ± 4.6
CA at test (weeks), mean ± SD	7.5 ± 6.8	8.7 ± 4.4
**Sex**		
Female	16 (55%)	15 (54%)
Male	13 (45%)	13 (46%)
**Race**		
White	23 (79%)	21 (75%)
Black	5 (17%)	1 (3.5%)
More than 1 race	1 (3%)	5 (18%)
Unknown race	−	1 (3.5%)
**Delivery method**		
Vaginal	9 (31%)	18 (71%)
Cesarean	20 (69%)	7 (25%)
Unknown	−	1 (4%)
Birth weight (grams), mean ± SD	1681 (664)	3652 (817)
SGA	6 (20%)	−
LGA	3 (10%)	5 (18%)
**Obstetric complications**		
Third-trimester bleeding	4 (14%)	−
Hypertensive disease of pregnancy	11 (38%)	1 (4%)
Rupture of membranes	6 (21%)	2 (7%)
Fetal growth restriction	6 (21%)	−
Breech position	3 (10%)	3 (11%)
Maternal substance use	2 (7%)	1 (4%)
Respiratory issues	23 (79%)	12 (43%)
Other	9 (31%)	5 (18%)
Antenatal steroid administration	21 (75%)	−
Retinopathy of prematurity	11 (38%)	−
APGAR at 5 min, median (min, max)	7 (2, 8)	8 (6, 9)*

All data were extracted from hospital records when available (29 preterm; 24 term), or self-report (0 preterm; 3 term). Data for 1 term infant was not provided. Data are expressed as sample size unless otherwise stated. GA, gestational age; PMA, postmenstrual age; CA, chronological age; SGA, small for gestational age, defined as < 10th percentile; LGA, large for gestational age, defined as > 90th percentile. Other obstetric complications include amniotic and placental complications, maternal fever/obesity/metabolic disease, fetal tachycardia, hypotonia, and nuchal cord.

*Apgar scores were not available for 4 term infants.

### Mice

**FIGURE 2:**
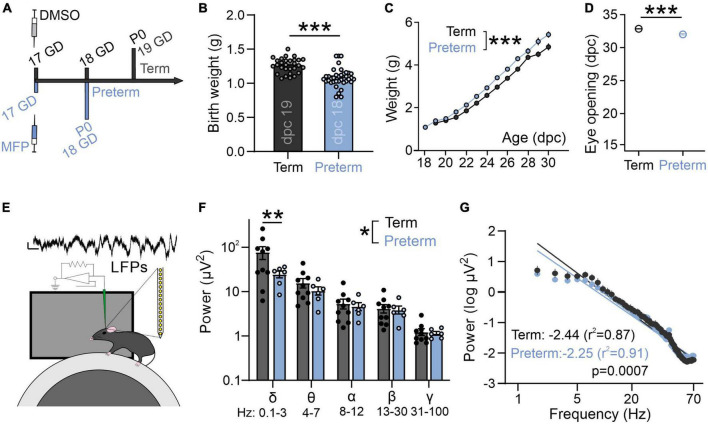
Preterm mice display a flatter slope of aperiodic LFP component. **(A)** Preterm mice are generated through the injection of Mifepristone (dissolved in DMSO) to pregnant dams at postconceptional day 17. Dams deliver pups within 24 h (1 day early: postnatal day/P 0). Term controls are generated by injecting pregnant dams at postconceptional day 17 with DMSO. **(B)** Preterm pups show a significantly lower birth weight [*N* = 29 term and 37 preterm mice, *t*-test, *t*(64) = 5.63, *p* < 0.0001] and **(C)** an accelerated postnatal growth rate [ordinary two-way ANOVA, *F*(1,433) = 98.53, *p* < 0.0001; *N* = 29 term and 19 preterm mice] and **(D)** precocious eye opening [*t*-test, *t*(25) = 7.95, *p* < 0.0001; *N* = 16 term and 11 preterm mice]. **(E)** Schematics of *in vivo* electrophysiology in awake mice. Local field potentials (LFPs) are collected using linear silicone probes (Neuronexus). Scale bar: 10 μV and 0.1 s. **(F)** Preterm birth significantly affects the distribution of power across different frequency bands [two-way ANOVA, *F*(4,56) = 2.568, *p* = 0.048; *N* = 6 preterm and 10 term and mice]. **(G)** Preterm mice show a significantly flatter slope of log transformed 2–70 Hz power. Linear regression; slope and *r*^2^ values are indicated. Data shown as mean or mean ± SEM. *p ≤ 0.05; **p ≤ 0.001; ***p ≤ 0.0001.

Mice were maintained on C57BL/6 background (The Jackson Laboratory, Bar Harbor, ME, USA) on standard 12:12 light:dark cycle, with food and water *ad libitum*. Animals from both sexes were used during the 4th week after birth. Preterm mice were generated through timed breedings, where the day after the pairing was considered as gestational day (GD) 0. Once the pregnancy was confirmed (>1.5 g increase in weight at GD 10), pregnant dams were habituated to handlers by daily handling. Mifepristone (MFP, Millipore Sigma, Burlington, MA, USA) was dissolved in DMSO and 150 μg was injected subcutaneously on GD 17 (Figure 2A; Dudley et al., 1996). Preterm mice were delivered on GD 18 (0.75–1 day early depending on the precise parturition time). Preterm mice have a significantly lower birth weight (Figure 2B), and increased mortality rates due to hypothermia, hypoxia, and inability to suckle (1–3 pups/litter). The cage with preterm mice was therefore supplemented with external heat and occasional oxygen to prevent hypothermia and hypoxia. Surviving pups are otherwise viable and display a catch-up growth typical of preterm infants (Figure 2C; Altigani et al., 1989). Preterm mice open their eyes significantly earlier, further suggesting accelerated development of visual brain areas after preterm birth (Figure 2D). Control term mice were obtained from timed pregnant dams injected with DMSO only on GD 17. To match the infant cohort as best as possible, all mice were tested in their fourth week of postnatal development as that period represents the onset of the critical period for binocular maturation in mice (Wang et al., 2010). Animals were treated in accordance with the University of Virginia Institutional Animal Care and Use Committee guidelines.

### *In vivo* electrophysiology in mice

V1 recordings were performed on awake term (*N* = 9) and preterm (*N* = 6) female and male mice, ages 21–28 days after birth, using a treadmill and blank screen as described in Niell and Stryker (2010). See Supplementary material for additional details.

### Immunohistochemistry and imaging

Brains from term and preterm mice (aged 35–40 days, *N* = 3–6/group, as indicated in text and figure legends) were sectioned into 40 μm, stained, and mounted on glass slides. Images were acquired from 4 to 6 sections/mouse (minimum 20 images/mouse). Quantification was performed on background subtracted images using ImageJ. See Supplementary material for additional details.

### Quantification and statistical analysis

Statistical analyses were performed blind to condition in GraphPad Prism 9.0 (GraphPad Inc., La Jolla, USA) using nested *t*-test and one or two-way ANOVA with *post-hoc* comparisons (as indicated in text and figure legends), unless stated otherwise. Spectral analysis was performed with Spike2 on 60 s of data at 500 Hz for both infant and mouse datasets. Power was normalized to the power in high frequency band (150–250 Hz). For infant data, power between 58 and 62 Hz was excluded to eliminate contamination from line noise which was present in some samples. Power spectral density values were averaged across channels of interest (Figure 1A). 1/f slope was generated using linear regression from 2 to 25 Hz for infants and 2–70 Hz for mice after log-transform of frequency and power. All data are reported as mean ± SEM, where N represents number of animals and infants used, unless indicated otherwise. Target power for all sample sizes was 0.8 and alpha was set to 0.05.

## Results

Neurocognitive and visual deficits of unclear origin are common in preterm children (Spierer et al., 2004; Edmond and Foroozan, 2006; Weinstein et al., 2012; Macintyre-Béon et al., 2013; Emberson et al., 2017; Chokron et al., 2021), and they are often diagnosed in school age, precluding early interventions (Ben Amor et al., 2012; de Kieviet et al., 2012; Fazzi et al., 2012). To determine how premature birth affects the development of neural activity at a circuit level, we used a cross-species approach to determine the properties of resting activity in a cohort of preterm infants and corrected age-matched term infants, and in a group of preterm mice and their term counterparts.

### Preterm infants and mice display accelerated maturation of resting activity in the visual cortex

We first used EEG in infants to measure resting state activity in the occipital and parietal visual areas within the first 4 months of life. While the preterm infants had a typical distribution of high power in low frequencies and low power in high frequencies (Figures 1B, C), power in theta and alpha bands was significantly reduced in preterm infants [Figure 1B; Two-way ANOVA frequency × birth interactions *F*(4,220) = 2.55, *p* = 0.039; Sidak’s multiple comparison test delta *p* = 0.43, theta *p* < 0.0001, alpha *p* = 0.02, beta *p* = 0.95, gamma *p* = 0.99].

As visual function matures earlier in preterm infants (Jandó et al., 2012), we then asked if the electrophysiological activity of the visual cortex would reflect this accelerated maturation. To test this, we calculated the slope of aperiodic EEG component 1/f (Figure 1C). 1/f is thought to reflect the background activity of the brain (Chini et al., 2022; Gyurkovics et al., 2022), and 1/f slopes become progressively flatter during infancy (Schaworonkow and Voytek, 2021). As previously reported (Schaworonkow and Voytek, 2021), we found that the power spectra of term and preterm infants were largely aperiodic (Figure 1C). However, we found that preterm infants had a significantly flatter 1/f slope when compared to term infants [*F*(1,1364) = 137.00, *p* < 0.0001], despite preterm infants being significantly younger in postmenstrual age [*t*(55) = −6.66, *p* < 0.001] and equivalent in chronological age [*t*(55) = −0.75, *p* = 0.455, see also Table 1]. 1/f slope was not correlated with postmenstrual age in term infants (Figure 1D), unlike in the preterm cohort, where a significant correlation between postmenstrual age and 1/f slope values was detected (*r*^2^ = 0.39, *p* = 0.01; Figure 1E). We detected an association [One-way ANOVA, *F*(4,52) = 14.86, *p* < 0.0001] between slope values and the degree of prematurity, with extremely (<28 gestational weeks at birth) and very (28–32 gestational weeks at birth) preterm infants showing the flattest 1/f slopes. We found no effects of mode of delivery on 1/f slope values in either term or preterm cohort [Table 1, C-section vs vaginal *t*-test; term *t*(26) = 0.19, *p* = 0.84; preterm *t*(27) = 0.1, *p* = 0.92], nor of respiratory issues reported in both cohorts [Table 1; term, *t*(26) = 0.57, *p* = 0.52; preterm *t*(27) = 0.1, *p* = 0.91]. Within the preterm cohort, we further found no relationship between the 1/f slope values and retinopathy of prematurity [*t*(27) = 0.3, *p* = 0.75], or between 1/f slope values and administration of antenatal steroids [*t*(27) = 1.5, *p* = 0.28], two most common complications in our cohort (Table 1). These results confirmed accelerated maturation of visual areas in preterm infants (Jandó et al., 2012; De Asis-Cruz et al., 2020) and that the timing of birth is a trigger for maturation of sensory brain areas (Toda et al., 2013).

To confirm whether electrophysiological activity of visual areas in a mouse model of preterm birth recapitulates changes seen in preterm infants (Figure 1C), we used *in vivo* electrophysiology to record intracortical local field potentials (LFPs) in layer 2/3 of the primary visual cortex (V1) of awake, young term and preterm mice (Figure 2E; Ribic et al., 2019). There was a significant interaction between the timing of birth and the energy composition of the power spectra [Figure 2F; Two-way ANOVA, frequency band x birth interaction *F*(4,52) = 2.93, *p* = 0.03], and *post-hoc* tests revealed a significant reduction of power in the delta band (Figure 2F, Sidak’s multiple comparison test delta *p* = 0.001, theta *p* = 0.99, alpha, beta and gamma *p* > 0.99). However, preterm mice also had a significantly flatter 1/f slope [Figure 2G; *F*(1,1061) = 11.51, *p* = 0.0007], indicating accelerated maturation of the primary visual cortex and suggesting the relative conservation of the effects of prematurity on neural activity in mice and humans.

### Preterm mice display elevated inhibition in visual cortex

**FIGURE 3:**
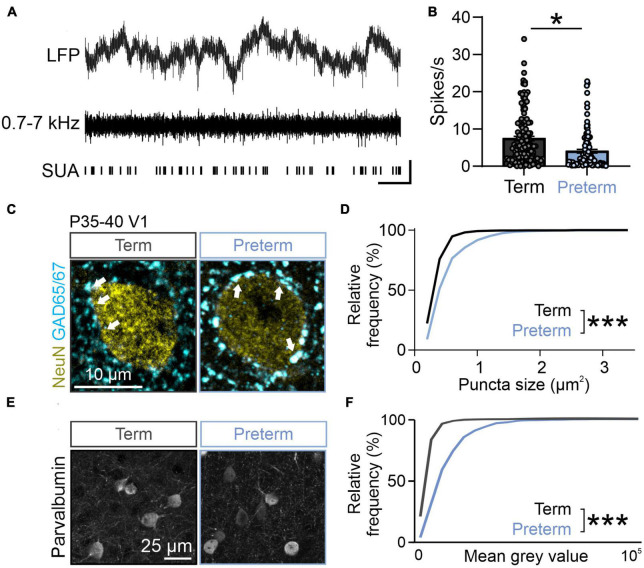
Preterm mice show elevated inhibition in the visual cortex. **(A)** Top: raw LFP trace; middle: filtered 0.7–7 kHz LFP; bottom: identified single units. **(B)** Preterm mice have a significantly reduced firing rate of neurons in the visual cortex during the presentation of a blank gray screen. 239 units from *N* = 5 term and 6 preterm mice, nested *t*-test, *t*(9) = 2.428, *p* = 0.038. Data shown as mean or mean ± SEM. **(C)** Immunohistochemical detection of NeuN (yellow) and GAD65/67 (cyan) in the visual cortex of term and preterm mice. Arrowheads: putative inhibitory synapses. **(D)** Cumulative frequency distribution of puncta size in term and preterm mice shows a significant shift to the right in term mice, signifying increased puncta size across all synaptic populations. Kolmogorov–Smirnov test, *p* < 0.001. Minimum 100 NeuN cell bodies/mouse from *N* = 5 preterm and 6 term mice. **(E)** Representative maximum projections of brain sections stained for Parvalbumin and quantified for Parvalbumin intensity. **(F)** Cumulative distribution of Parvalbumin intensity measurements from. The curve in preterm mice is shifter to the right, demonstrating the significantly increased intensity of Parvalbumin staining in preterm mice. *N* = 477 and 636 cell bodies from 3 preterm and 6 term mice, respectively. Kolmogorov–Smirnov test, *p* < 0.001. *p ≤ 0.05; ***p ≤ 0.0001.

The fourth postnatal week represents a critical transition toward visually-driven activity in mice, reflected in increased suppression of spontaneous neuronal activity by rising levels of inhibitory neurotransmission (Toyoizumi et al., 2013; Shen and Colonnese, 2016). Given the flatter, “older” 1/f slope in preterm mice (Figures 2F, G), we hypothesized that the spontaneous firing rates of visual cortex neurons would be lower in preterm mice reflecting accelerated transition to visually-driven activity. We isolated firing profiles of neurons from all layers of the cortex (Figure 3A) and estimated their firing rates in stationary, awake mice whose eyes were centered on a blank, gray screen (Figure 2E). We indeed found a significantly reduced spontaneous firing rate of visual cortex neurons in preterm mice [Figure 3B; nested *t*-test *t*(9) = 2.42, *p* = 0.030; 239 isolated neurons from 5 term and 6 preterm mice].

Suppressed spontaneous firing could be a result of increased inhibition in the developing cortex (Toyoizumi et al., 2013; Shen and Colonnese, 2016; Ribic, 2020). To test this and probe the cellular mechanism of reduced spontaneous firing in preterm mice, we quantified the expression of inhibitory synapse marker glutamic acid decarboxylase 65/67 (GAD65/67) using quantitative immunohistochemistry (Chattopadhyaya et al., 2007; Carcea et al., 2014; Gogolla et al., 2014). GAD65/67 is detected as punctate labeling that surrounds neuronal cell bodies, in line with its localization in the perisomatic inhibitory synapses (Chattopadhyaya et al., 2007). The number and size of perisomatic GAD65/67 puncta are a reliable indicator of inhibitory neurotransmission levels (Carcea et al., 2014). In agreement with suppressed spontaneous activity due to increased inhibition in preterm visual cortex, the size of perisomatic GAD65/67 puncta was significantly increased in preterm mice (Figures 3C–E; Kolmogorov–Smirnov test *p* < 0.0001, *D* = 0.38), with no changes in their density (Term = 15.62 ± 0.4, Preterm = 14.98 ± 0.88 puncta/100 μm^2^ of NeuN+ soma; *N* = 6 and 5 mice). Cortical perisomatic inhibition is mediated by fast-spiking, Parvalbumin-expressing interneurons (Pfeffer et al., 2013). As previous studies of preterm birth-related brain injury models reported changes in Parvalbumin interneuron distribution and density (Panda et al., 2018; Stolp et al., 2019; Scheuer et al., 2021), we then asked if this neuronal population is affected by preterm birth. Cortical interneurons represent a mixture of high, middle and low Parvalbumin (PV)-expressing interneurons (Donato et al., 2013), with low PV interneurons being the dominant group in the developing brain and high PV in the mature brain (Donato et al., 2013). In agreement with accelerated maturation of the visual cortex, preterm mice had a significantly shifted distribution of PV intensities, with a higher proportion of middle and high PV-expressing interneurons (Figures 3E, F; Kolmogorov–Smirnov test *D* = 0.45, *p* < 0.0001), without any changes in the overall density of PV interneurons (Term = 22.45 ± 3.53, Preterm = 23.79 ± 2.56 PV interneurons per field of view, *N* = 5 term and 3 preterm mice). Altogether, these results demonstrate accelerated maturation of the visual cortex after preterm birth and suggest a central role of inhibition in this process.

## Discussion

Despite extensive research, effects of premature birth on cortical activity in the early postnatal period remain unclear. Through a comparative approach, our study identified accelerated maturation of neural activity in the visual cortex of preterm infants and mice driven by elevated levels of inhibition.

Cortical oscillatory activity shows distinct developmental patterns, with a reduction in the relative power of low frequencies and an increase in high frequencies with increasing age (Gasser et al., 1988; Tierney et al., 2013). Such distribution of powers is likely responsible for the age-related flattening of 1/f EEG slope (Saby and Marshall, 2012; Schaworonkow and Voytek, 2021). An “older” spectral profile in preterm infants and mice is in agreement with the notion that premature exposure to extrauterine environment accelerates brain maturation, at least in primary sensory areas such as the visual cortex (Jandó et al., 2012; De Asis-Cruz et al., 2020). Interestingly, flattened 1/f slope is found in children diagnosed with attention deficit hyperactivity disorder (ADHD), a condition with high prevalence in the preterm population (Johnson and Marlow, 2011; Ostlund et al., 2021; Arnett et al., 2022). Future studies can address if flatter 1/f slope distinguishes preterm-born children with and without ADHD. As primary sensory areas mature earlier than the frontal executive and association areas (Reh et al., 2020; Sydnor et al., 2023), future studies can determine if the accelerated maturation of sensory areas dysregulates the sequence of cortical maturation, impairing the functional maturation of frontal brain areas. This is particularly important given the high prevalence of executive function and visual attention disorders in preterm children (Arpino et al., 2010; Johnson and Marlow, 2011; Burstein et al., 2021).

Fast-spiking, Parvalbumin interneurons are central for the maturation of cortical circuits (Reh et al., 2020; Ribic, 2020). Their functional development is sensitive to experience and in the visual cortex, their maturation can be accelerated or delayed through manipulations of visual input (Komitova et al., 2013; Ye and Miao, 2013; Guan et al., 2017). In agreement, our results suggest that premature onset of visual input can accelerate the maturation of cortical Parvalbumin interneurons, shifting their distribution to mid- and high-PV expressing interneurons in preterm mice. Previous studies of hypoxic mouse models of preterm birth have demonstrated that hypoxia results in reduced density of Parvalbumin interneurons, as well as a decrease in the intensity of Parvalbumin signal (Komitova et al., 2013). The differing findings in our study are potentially due to low or absent hypoxia in our mouse model, as well as differences in how preterm brain injury is modeled. Hypoxia models of preterm birth are commonly term-born, with continuous or intermittent exposure to hypoxia during the postnatal development (van der Kooij et al., 2010; Komitova et al., 2013; Salmaso et al., 2014; Lacaille et al., 2019). While hypoxia represents a severe injury, it may not recapitulate the effects of preterm birth alone. Birth itself is an environmental shock that can significantly affect neuronal and synaptic development and accelerate developmental trajectories of different brain areas (Toda et al., 2013; Castillo-Ruiz et al., 2020). As the effects of preterm birth alone on sensory development are unclear, our results highlight a need for multiple animal models to capture the variability in the degree of preterm birth-related brain injury.

Another potential cause of divergence between our findings in preterm mice and previously published findings on interneuronal populations (Lacaille et al., 2019, 2021) in preterm infant cortex is the degree of prematurity. The most vulnerable population of preterm infants are born extremely early, prior to week 28 of gestation, and very early (28–32 weeks of gestation). These are also the infants that show deficits in cortical interneurons (Tibrewal et al., 2018; Lacaille et al., 2019, 2021; Stolp et al., 2019). Yet, the majority (>70%) of preterm infants are born moderately to late preterm (32–37 weeks of gestation), with variable degrees of health complications, including hypoxic brain injury (Shapiro-Mendoza and Lackritz, 2012; Karnati et al., 2020). Infants in our study reflect this, with 51.7% born moderately to late preterm (Table 1). Considering the relatively high viability of preterm mice, as well as our finding of flattest slopes in extremely and very preterm infants, our results suggest that mice born a day early are a model of middle to late preterm birth. However, as both early and late preterm infants have poor neurocognitive outcomes (Arpino et al., 2010; Ben Amor et al., 2012; Karnati et al., 2020; Crump et al., 2021), our study further confirms that birth timing alone is sufficient to dysregulate the maturational trajectory of the brain.

Term-born mouse pups in the first postnatal week are commonly compared to preterm newborns, based on cortical development milestones (Colonnese et al., 2010; Semple et al., 2013). While direct comparison between developmental stages of mice and humans is difficult due to different rates of maturation, our results from the mouse sample confirm previous findings of accelerated brain maturation after premature birth (Toda et al., 2013; Castillo-Ruiz et al., 2020), and provide evidence for it in preterm infants (De Asis-Cruz et al., 2020). Our results further highlight the utility of EEG measures in the clinical setting and set the stage for future longitudinal studies that will explore the relationship between 1/f slope and neurodevelopmental outcomes in the preterm population. Our study adds to the growing body of evidence that birth itself is a critical transition during brain development. Future studies will explore how the timing of this transition affects sensory and cognitive processing, given that preterm infants are at higher risk for developing neurodevelopmental and neuropsychiatric disorders (Johnson and Marlow, 2011).

## Data availability statement

The raw data supporting the conclusions of this article will be made available by the authors, without undue reservation.

## Ethics statement

The studies involving human participants were reviewed and approved by the University of Virginia Institutional Review Board for Health Sciences Research. Written informed consent to participate in this study was provided by the participants’ legal guardian/next of kin. This animal study was reviewed and approved by the University of Virginia Animal Care and Use Committee.

## Author contributions

AR and MP conceived and designed the study. AR acquired and analyzed mice data, analyzed infant data, and wrote the manuscript with input from all authors. MP, MN, WC, AB, and AD acquired infant data. MP analyzed infant data. EM analyzed infant and mice data. IW, TH, and CS collected and analyzed the immunohistochemistry data. All authors contributed to the article and approved the submitted version.
